# Supplementary material for: Pathogenesis-Targeted Preventive Strategies for Multidrug Resistant Ventilator-Associated Pneumonia: A Narrative Review
Source: Microorganisms. 2020 May 30;8(6):821. doi: 10.3390/microorganisms8060821 (PMC7356213; doi:10.3390/microorganisms8060821)
Supplement: Supplementary file 1 [file microorganisms-08-00821-s001.zip › Supplementary materials Revisione VAP MDR PREV/Table 2 review results v1.2.docx]

**Table 2.** Summary of the results of the studies included in our narrative review, grouped by the type of preventive strategy

| **Preventive strategy** | **Study** | **Study design** | **Outcomes of interest** | **Comments** |
| --- | --- | --- | --- | --- |
| Oral hygiene with CHX | Tuon et al. (2017) | RCT | Day 5, all patients had a positive culture result for the OM and DP including ESBL, CRPA, KPC, MRSA, CRAB  CHX resulted in significant reduction in the total number of MDR bacteria and percentage of MRSA  High susceptibility of *S. aureus* and Gram-negative bacilli for CHX, even after brief exposure and relatively low concentrations | Sample size too small to assess the influence of CHX on the incidence of MDR-VAP |
| CHX bathing and cleansing | Noto et al. (2015) | RCT | Non-significant difference in individual infections between 2% CHX impregnated and non-antimicrobial impregnated (aRR in treatment group: 0.94; 95% CI 0.65-1.37; p=0.83)  Three different post-hoc analyses resulted in a significant increase in possible or probable VAP with the use of 2% CHX-impregnated cloths  No difference in the rate of cultures positive for MDR organisms between the two bathing periods | Small absolute number of VAP events compared to the number of patients may have avoided a to explore for any infection-specific differences in the positivity of cultures for MDR between the two bathing periods |
|  | Boonyasiri et al. (2016) | RCT | No significant difference in number of MDR-related favorable events and incidence rates of VAP between the use of 2% CHX and non-antimicrobial impregnated washcloths (6.5 vs 6.1 episodes per 1000 ventilator-days, p=0.69)  No significant difference in MDR-likely etiologies of VAP between the two cleansing strategies |  |
| Selective digestive decontamination | Pèrez-Granda et al. (2018) | Prospective cohort | Significant decrease in VAP rate after the introduction of SDD both in overall study population (16.26/1,000 days and 6.80 episodes/1,000 days of MV, p=0.01) and high-risk patients (25.85/1,000 days and 12.06 episodes/1,000 days of MV, p=0.04 )  Delayed occurrence of VAP among the patients receiving SDD  SDD use was able to reduce the number of isolates positive for MDR or XDR P. aeruginosa (p<0.001) with no increase in the total number of MDR pathogens |  |
|  | Sánchez-Ramírez et al. (2018) | Prospective cohort | Significant reduction in MDR-infections, VAP included, with the use of SDD (RR 0.43; 95% CI 0.32–0.59, p<0.001)  Significant decrease in antibiotic consumption  Non-significant increase in adjusted rate of ICU colonization resistance |  |
| Multiple decontamination regimens | Camus et al. (2015) | Prospective cohort | Decrease in all-cause ICU-acquired infection with a combination of multiple decontamination regimens  Three-fold decline in total intubation-related pneumonias  Statistically significant decrease in P. aeruginosa (1.7/1000 vs 3.5/1000).*,* MDR *Enterobacteriaceae* and CRGNB related infections  Non-significant decrease in *MRSA* infection rates |  |
| Probiotic preparation | Mahmoodpoor et al. (2019) | RCT | Significant decrease in the incidence of microbiologically confirmed VAP, hospital and ICU stay and gastric residuals  Non-significant decrease in MDR colonization and time to first VAP | The composition of the study population and the microbiological criteria for the diagnosis of VAP may have influenced the results |
| Silver-coated endotracheal tube | Afessa et al. (2010) | Retrospective cohort | Significant mortality reduction in patients diagnosed with VAP in the ETT group (14% vs 36%, p=0.03)  50% lower rate of VAP by potentially MDR bacteria in the ETT group |  |
| Universal gloving and contact isolation | Bearman et al. (2007) | RCT | Increased nosocomial infection rates during universal gloving periods, VAP included (0 vs 2.3 episodes per 1000 device days, p<0.001)  No difference in *MRSA* and *VRE* colonization rates between the two study periods | Inferior compliance relative to hand hygiene was noted during the universal gloving period |
|  | Bearman et al. (2010) | Prospective cohort | No difference in device-associated infection incidence rates, VAP included (1.0 vs 1.1 cases/1000 device days, p=0.09)  No difference in *MRSA* and *VRE* colonization rates between the two study periods | The study was undertaken after aggressive hospital-wide hand-hygiene education |
|  | Zahar et al. (2013) | Retrospective cohort | Significant increased incidence of MDR-VAP in isolated patients (sHR 2.1, 95% CI 1.3–3.3, p=0.002), along with the risk of medical errors and other non-infectious adverse events |  |
| Alcohol-based hand gel | Rupp et al. (2008) | RCT | No significant relationship with VAP rates and infections due to *MRSA*, *VRE* and *C. difficile*  Rings, fingernails length < 2 mm and lack of access to hand gel were all associated to increased microbial carriage on hands |  |
| Environmental decontamination with VHP | Ray et al. (2008) | Prospective cohort | VHP was effective to stop an outbreak of MDR *A. baumannii*, which most commonly caused VAP in a LTACH | LTACH setting and sample size |
| Bundles of care | Wilks et al. (2006) | Prospective cohort | A structured bundle of control measures to improve both hand hygiene and patient care was effective to stop an outbreak of MDR A. baumannii |  |
|  | Berriel-Cass et al. (2006) | Prospective cohort | Significant decrease in ICU length-of-stay, total ventilator days and VAP rate (from the average of 8.2 per 1000 ventilator days for 13 months to 3.3 per 1000 ventilator days for 24 months, p=0.02) |  |
|  | Landrum et al. (2008) | Prospective cohort | Aggressive implementation of aggressive wide-ranging infections control interventions led to a marked reduction in VAP rates (from 60.6 to 11.1 per 1000 ventilator days, p=0.029), with sustained improvement  Sustained improvement in antimicrobial susceptibility of *A. baumannii* (the most common pathogen causing VAP in that setting) | Military field setting |
|  | Walkey et al. (2009) | Prospective cohort | A bundle of interventions based on CDC guidelines resulted in 56% reduction in VAP rate (95% caused by MDR germs) (from 3.8 to 1,67 cases per 1000 ventilator-days after the implementation of the VAP-bundle approach, p<0.001) | LTACH setting |
|  | Khan et al. (2009) | Case-control | Non-significant decrease in VAP rates (18% to 13%, p=0.11) | Lack of an established infrastructure for infection control |
|  | Alp et al. (2014) | Retrospective cohort | Non-significant decrease in VAP rates (incidence rate ratio, IRR = 0.88, p=0.574)  Significant reduction in MRSA, P. aeruginosa and A. baumannii colonization (IRR 0.13, p<0.001 after 3 years; IRR 0.63, p=0.002 after 8 years and IRR 0.53, p<0.001 after 8 years respectively) |  |
|  | Righi et al. (2014) | Retrospective cohort | Significant reduction in VAP incidence (from 15.9% to 6.7%, p<0.001), both early- and late-onset VAP (6.6% to 1.9%, p<0.001 and 9.3% to 4.7%, p=0.001 respectively)  Significantly decreased risk of developing MDR-VAP after the implementation of SDD in the bundle (OR 0.54; 95% CI, 0.31-0.91). |  |
|  | Gao et al. (2015) | Prospective cohort | Decrease in VAP incidence (from 32.72/1000 to 24.60/1000) and in percentage of MDR pathogens (from 67.91% to 59.68%) |  |
|  | Ali et al. (2016) | Retrospective cohort | Progressive decrease in VAP incidence rates (5.42 per 1000 ventilator-days in 2010, 5.91 per 1000 ventilator-days in 2011, and 3.88 per 1000 ventilator-days in 2012) | Adherence rates to bundle have not been measured |
|  | Ibn Saied et al (2017) | Retrospective cohort | Decrease in early-onset VAP incidence rates | Adherence rates to bundle have not been measured |
|  | Khurana et al. (2017) | Prospective cohort | Inverse correlation between VAP incidence and compliance to ventilator bundle + hand hygiene |  |
|  | Dananché et al. (2018) | Prospective cohort | Decline in VAP incidence rates partially explained by improvement in ventilator bundle adherence (aIRR 95%CI 0.88, 0.82–0.94, p<0.001 and 0.89, 0.81–0.98, p=0.022, in the young and old group respectively)  Significant decrease in *MRSA-*related VAP incidence in all age groups  Stable and reduced *CRPA*-related VAP incidence in the young and old groups  Increase in *3GCRE*-related VAP incidence in all age groups | Adherence rates to bundle have not been measured |
|  | Kanafani et al. (2018) | Prospective cohort | Decreased MDR *A. baumannii* colonization pressure during a 2 year outbreak |  |
|  | Kanafani et al. (2019) | Retrospective cohort | Significant reduction in VAP incidence rates after the combined adoption of ventilator bundle + multiple aggressive IC control strategies (from 13.1 in 2008 to 1.1 per 1000 ventilator-days in 2017, with a reduction rate of 91.6%) |  |

3GCRE = 3^rd^ generation cephalosporines-resistant *Enterobacteriaceae*, aRR = adjusted risk ratio, CDC = Centre for Disease Control and Prevention, CHX = Chlorhexidine, CI = confidence interval, CRAB = carbapenem-resistant *Acinetobacter* baumannii, CRGNB = carbapenem-resistant Gram-negative bacilli, CRPA = carbapenem-resistant *Pseudomonas aeruginosa*, ESBL = extended-spectrum beta-lactamase, ETT = endotracheal tube, IC = infection control, ICU = intensive care unit, IRR = incidence rate ratio, LTACH = long-term acute care hospital, MDR = multidrug-resistant, MRSA = methicillin-resistant *Staphylococcus* aureus, RCT = randomized controlled trial, RR = risk ratio, SDD = selective digestive decontamination, sHR = sub-distribution hazard ratio, XDR = extensively drug-resistant, VAP = ventilator-associated pneumonia, VHP = vaporized hydrogen peroxide, VRE = vancomycin-resistant *Enterococci*
