# Supplementary material for: Pathogenesis-Targeted Preventive Strategies for Multidrug Resistant Ventilator-Associated Pneumonia: A Narrative Review
Source: Microorganisms. 2020 May 30;8(6):821. doi: 10.3390/microorganisms8060821 (PMC7356213; doi:10.3390/microorganisms8060821)
Supplement: Supplementary file 1 [file microorganisms-08-00821-s001.zip › Supplementary materials Revisione VAP MDR PREV/Table 1 recommendations v1.2.docx]

**Table 1.** Summary of the recommendations for ventilator-associated pneumonia prevention provided by major scientific societies.

| **SHEA/IDSA practice recommendation – 2014 update** | | | |
| --- | --- | --- | --- |
| Recommendation | Preventive strategy | Remarks |  |
| Basic practices | Use of NIPPV | Suggested for selected populations |  |
|  | Manage patients without sedation whenever possible |  |  |
|  | Interrupt sedation daily |  |  |
|  | Assess readiness to extubate daily |  |  |
|  | Perform SBT with sedatives turned off |  |  |
|  | Facilitate early mobility |  |  |
|  | ETTs with subglottic secretion drainage ports | For patients expected to require greater than 48 or 72 hours of mechanical ventilation |  |
|  | Change the ventilator circuit only if visibly soiled or malfunctioning |  |  |
|  | Head of bed elevated to 30-45° | There are very little data on head-of-bed elevation, but it is classified as a basic practice because of its simplicity, ubiquity, low cost, and potential benefit |  |
| Special approaches | Selective oral or digestive decontamination | There are abundant data on the benefits of digestive decontamination but insufficient data on the long-term impact of this strategy on antimicrobial resistance rates |  |
|  | Regular oral care with Chlorhexidine |  |  |
|  | Prophylactic probiotics |  |  |
|  | Ultrathin polyurethane ETT cuffs |  |  |
|  | Automated control of ETT cuff pressure |  |  |
|  | Saline instillation before tracheal suctioning |  |  |
|  | Mechanical toothbrushing |  |  |
| Generally not recommended | Silver-coated ETTs |  |  |
|  | Kinetic beds |  |  |
|  | Prone positioning |  |  |
|  | Stress ulcer prophylaxis | May be indicated for reasons other than VAP prevention |  |
|  | Early tracheostomy | May be indicated for reasons other than VAP prevention |  |
|  | Monitoring residual gastric volumes | May be indicated for reasons other than VAP prevention |  |
|  | Early parenteral nutrition | May be indicated for reasons other than VAP prevention |  |
| No recommendation | Closed/in-line endotracheal suctioning |  |  |

| **Spanish VAP prevention guidelines 2013** | | |
| --- | --- | --- |
| Recommendation | Preventive strategy |  |
| Basic mandatory | Education and training in appropriate airway management |  |
|  | Strict hand hygiene for airway management |  |
|  | Cuff pressure control |  |
|  | Oral hygiene with Chlorhexidine |  |
|  | Semi-recumbent positioning. Avoid 0°, if possibile |  |
|  | Procedures and protocols which safely avoid or reduce time on ventilator |  |
|  | Avoid scheduled change of ventilator circuit, humidifiers and endotracheal tubes |  |
| Highly recommended | Selective decontamination of the digestive tract or selective decontamination of the oropharynx |  |
|  | Aspiration of subglottic secretions |  |
|  | Short course of intravenous antibiotic |  |

|  | **SFAR – SLRF guidelines on hospital-acquired pneumonia in ICU 2017** | | | |  |
| --- | --- | --- | --- | --- | --- |
| Preventive strategy | | Remarks | | |  |
| Standardised multimodal HAP prevention approach | |  |  |  | |
| Routine selective digestive decontamination and maximal 5-day course of systemic prophylactic antibiotic | | In ICUs where MDR bacteria prevalence is low (<20%) | | |  |
| Within a standardised multimodal HAP prevention approach, the combination of some of the following methods are suggested:  Promote the use of non-invasive ventilation to avoid tracheal intubation*  Favor orotracheal over nasotracheal intubation  Limit dose and duration of sedatives an analgesics°  Initiate early enteral feeding (within the first 48 hours of admission)  Regularly verify endotracheal tube cuff pressure  Perform sub-glottic suction (every 6-8 hours) using an appropriate endotracheal tube | | * Mainly in post-operative digestive surgery and in COPD patients  ° Promote their use guided by sedation/pain/agitation scales and/or daily interruption | | |  |
| Within a standardised multimodal HAP prevention approach, the combination of some of the following methods are discouraged:  Systematic early (< day 7) tracheotomy*  Anti-ulcer prophylaxis*  Post-pyloric enteral feeding*  Administration of probiotics and/or synbiotics  Early systematic change of the humidifier filter°  Use of suctioning systems for endotracheal secretions  Use of antiseptic-coated intubation tubes or with tubes with an “optimized” cuff shape  Selective oropharyngeal decontamination (SOD) with povidone-iodine  Use of prophylactic nebulized antibiotics  Daily skin decontamination using antiseptics | | * Except for specific indications  ° Except for specific manufacturer recommendations | | |  |
| Use of non-invasive ventilation in weaning of COPD patients | |  | | |  |

|  | **The Intensive Care Society recommended bundle of interventions for the prevention of VAP 2016** |  |
| --- | --- | --- |
| Elevation of head of bed (30° – 45°) | | |
| Daily sedation interruption and assessment of readiness to extubate | | |
| Use of subglottic secretion drainage | | |
| Avoidance of scheduled ventilator circuit changes | | |

| **International ERS/ESICM/ESCMID/ALAT guidelines for the management of HAP and VAP 2017** | | |
| --- | --- | --- |
| Preventive strategy | Comments |  |
| Selective oral decontamination and/or selective digestive decontamination | These guidelines suggest the use of SOD, but not SDD, in settings of low-rates of antibiotic resistant bacteria and low antibiotic consumption |  |
| Selective oral decontamination with Chlorhexidine | The guideline panel decided not to issue a recommendation on the use of CHX to perform SOD in patients requiring mechanical ventilation until more safety data become available, due to the unclear balance between a potential reduction in pneumonia rate and a potential increase in mortality. |  |

ALAT = Latin American Thoracic Association, COPD = Chronic obstructive pulmonary disease, ERS = European Respiratory Society, ESCMID = European Society of Clinical Microbiology and Infectious Diseases, ESICM = European Society of Intensive Care Medicine, ETT = Endotracheal tube, HAP = Hospital-acquired pneumonia, NIPPV = Non-invasive positive pressure ventilation, SBT = Spontaneous breathing trial, SOD = Selective oropharyngeal decontamination, VAP = Ventilator-associated pneumonia.
