# Supplementary material for: Pathogenesis-Targeted Preventive Strategies for Multidrug Resistant Ventilator-Associated Pneumonia: A Narrative Review
Source: Microorganisms. 2020 May 30;8(6):821. doi: 10.3390/microorganisms8060821 (PMC7356213; doi:10.3390/microorganisms8060821)
Supplement: Supplementary file 1 [file microorganisms-08-00821-s001.zip › Supplementary materials Revisione VAP MDR PREV/Table 3.docx]

**Table 3.** Advantages and limitations of the preventive strategies addressed in our review

| **Preventive strategy** | **Advantages** | **Limitations** |
| --- | --- | --- |
| Oral hygiene with CHX | Limited cost  Easily feasible | May increase mortality  Potential allergic reactions  Transient oral lesions in some patients  Concerns about potential selection of resistant strains |
| CHX bathing and cleansing | Limited cost  Easily feasible  Variety of preparations and methods of application  Reduced skin bioburden | May increase mortality  Potential skin reactions  Concerns about potential selection of resistant strains |
| Selective digestive decontamination | Limited cost  Easily feasible  Reduced colonization pressure | Concerns about potential selection of resistant strains |
| Multiple decontamination regimens | Easily feasible  Reduced colonization pressure | Complexity |
| Probiotic preparation | Limited cost  Easily feasible  Pleiotropic effect  Variety of preparations | Concerns about bacteremia and fungemia in certain high risk groups  Appropriate preparation, dose and timing of administration difficult to be defined |
| Silver-coated endotracheal tube | Easily feasible | Higher acquisition cost  Availability until tracheostomy |
| Universal gloving and contact isolation | Limited cost | Adherence required  Potential decrease in hand hygiene |
| Alcohol-based hand gel | Limited cost | Adherence required |
| Environmental decontamination with VHP | Wide surfaces disinfection | Temporary environmental unavailability  Need of appropriate devices |
| Bundles of care | Integrated protocols  Adaptability | Complexity  Need of advanced staff education |

CHX = Chlorhexidine, VHP = vaporized hydrogen peroxide
