# Supplementary material for: Pathogenesis-Targeted Preventive Strategies for Multidrug Resistant Ventilator-Associated Pneumonia: A Narrative Review
Source: Microorganisms. 2020 May 30;8(6):821. doi: 10.3390/microorganisms8060821 (PMC7356213; doi:10.3390/microorganisms8060821)
Supplement: Supplementary file 1 [file microorganisms-08-00821-s001.zip › Supplementary materials Revisione VAP MDR PREV/Flowchart PRISMA 2009 v1.2.doc]

**Screening**

**Included**

**Eligibility**

**Identification**

Records identified through database searching
(n = 225)

Records after duplicates removed
(n = 178)

Records screened
(n = 178)

Full-text articles assessed for eligibility
(n = 50)

Full-text articles excluded
(n = 23)

23 non relevant

Studies included in qualitative synthesis
(n = 27)

Records excluded
(n = 128)

57 reviews, 1 position paper, 4 congress presentations, 1 editorial, 2 guidelines, 2 trial protocols, 11 non-human studies, 2 non-English publications, 48 non-relevant

**Figure S1.** Flowchart of the selected studies following PRISMA 2009 (Preferred Reporting Items for Systematic reviews and Meta-Analyses) guidelines.
